# Exosome-based drug delivery systems for the treatment of diabetes and its complications: current opinion

**DOI:** 10.20517/evcna.2023.32

**Published:** 2023-09-04

**Authors:** Qi Chen, Jie Chen, Yi-Ning Liu, Su-Hua Qi, Lin-Yan Huang

**Affiliations:** ^1^Xuzhou Key Laboratory of Laboratory Diagnostics, School of Medical Technology, Xuzhou Medical University, Xuzhou 221004, Jiangsu, China.; ^2^Pharmacology College, Xuzhou Medical University, Xuzhou 221004, Jiangsu, China.; ^#^Authors contributed equally.

**Keywords:** Exosomes, drug carrier, diabetes, diabetic complications

## Abstract

Diabetes medication is based on controlling blood glucose and delaying the onset of related complications and is not a complete cure for diabetes. Conventional drug therapy fails to stop progressive islet β cell failure in diabetic patients. Recent studies have shown that "exosome-based therapy" holds great promise in treating diabetes and its complications. Exosomes are small vesicles that are stable in the bloodstream and can effectively deliver therapeutic drugs to specific tissues or organs through intercellular communication. Using exosomes as carriers for drug delivery offers several advantages. This review summarizes the benefits of exosomal drug delivery systems, drug loading methods, and their applications in treating diabetes and its complications. However, there are still challenges to overcome in using exosomal drug delivery systems, such as large-scale production, assessing the contents of exosomes, and monitoring the safety and effectiveness of the treatment *in vivo*. In conclusion, this review proposes the therapeutical potential of exosomes as drug carriers for developing novel drugs to provide new strategies for treating diabetes and its complications.

## INTRODUCTION

Diabetes mellitus (DM) is a chronic metabolic disease characterized by the destruction of pancreatic β cells by the immune system^[1,2]^, leading to impaired insulin secretion or reduced insulin sensitivity^[3]^. Consequently, this condition results in persistently elevated blood glucose levels, known as hyperglycemia. According to the International Diabetes Federation (IDF), the number of individuals worldwide living with diabetes is projected to reach 537 million by 2023, and is expected to rise to 783 million by 2045^[4]^. Diabetes has emerged as one of the most rapidly growing global health crises in the 21st century, and prolonged exposure to high glucose levels can lead to organ damage and dysfunction^[5]^. Common complications associated with diabetes include microvascular damage, diabetic retinopathy, diabetic nephropathy, and peripheral neuropathy.

Achieving effective diabetes control necessitates adherence to several fundamental principles, including making healthier dietary choices, engaging in regular exercise, strictly following prescribed medications, continuously monitoring blood glucose levels, and acquiring knowledge on how to maintain optimal health^[6]^. Furthermore, research has demonstrated that medication alone has limited efficacy without lifestyle interventions. Therefore, personalized treatment and diabetes control education are paramount and should be prioritized^[7]^. By adhering to these principles, individuals with diabetes can effectively manage their condition and significantly enhance their overall health and well-being^[8]^. Currently, available treatments for diabetes encompass insulin injections and oral hypoglycemic agents. However, these treatments may give rise to side effects such as hypoglycemia, anxiety, fatigue, and loss of consciousness^[9]^. Similarly, existing treatments for diabetic complications are also burdened with side effects and other challenges. Consequently, it is imperative to explore novel therapeutic agents and treatment strategies for diabetes and its complications.

Exosomes have emerged as a promising approach for the treatment of diabetes and its associated complications. These vesicles possess several advantageous properties, including immunocompatibility^[10]^, low immunogenicity, high stability in the bloodstream^[11]^, and the ability to facilitate targeted drug delivery. Moreover, they evade rapid clearance by monocytes and macrophages upon entering the body and exhibit an enhanced osmotic retention effect^[12,13]^. Exosomes facilitate intercellular communication through autocrine, paracrine, and endocrine signaling, thereby exerting their effects on neighboring cells^[14,15]^. Their inherent structure and characteristics make them an ideal vehicle for delivering drugs to specific tissues or organs^[16-18]^. Numerous studies have demonstrated the beneficial properties of exosomes in mitigating the detrimental effects of diabetes, such as impaired wound healing and peripheral neuropathy. Therefore, exosomes hold great promise as a novel therapeutic intervention to enhance the quality of life for individuals affected by diabetes. This review aims to provide an overview of the unique advantages of exosomes, drug delivery modalities, and recent advancements in exosome-based drug delivery for the treatment of diabetes and its complications.

## BIOLOGICAL CHARACTERISTICS AND FUNCTIONS OF EXOSOMES

**Figure 1:**
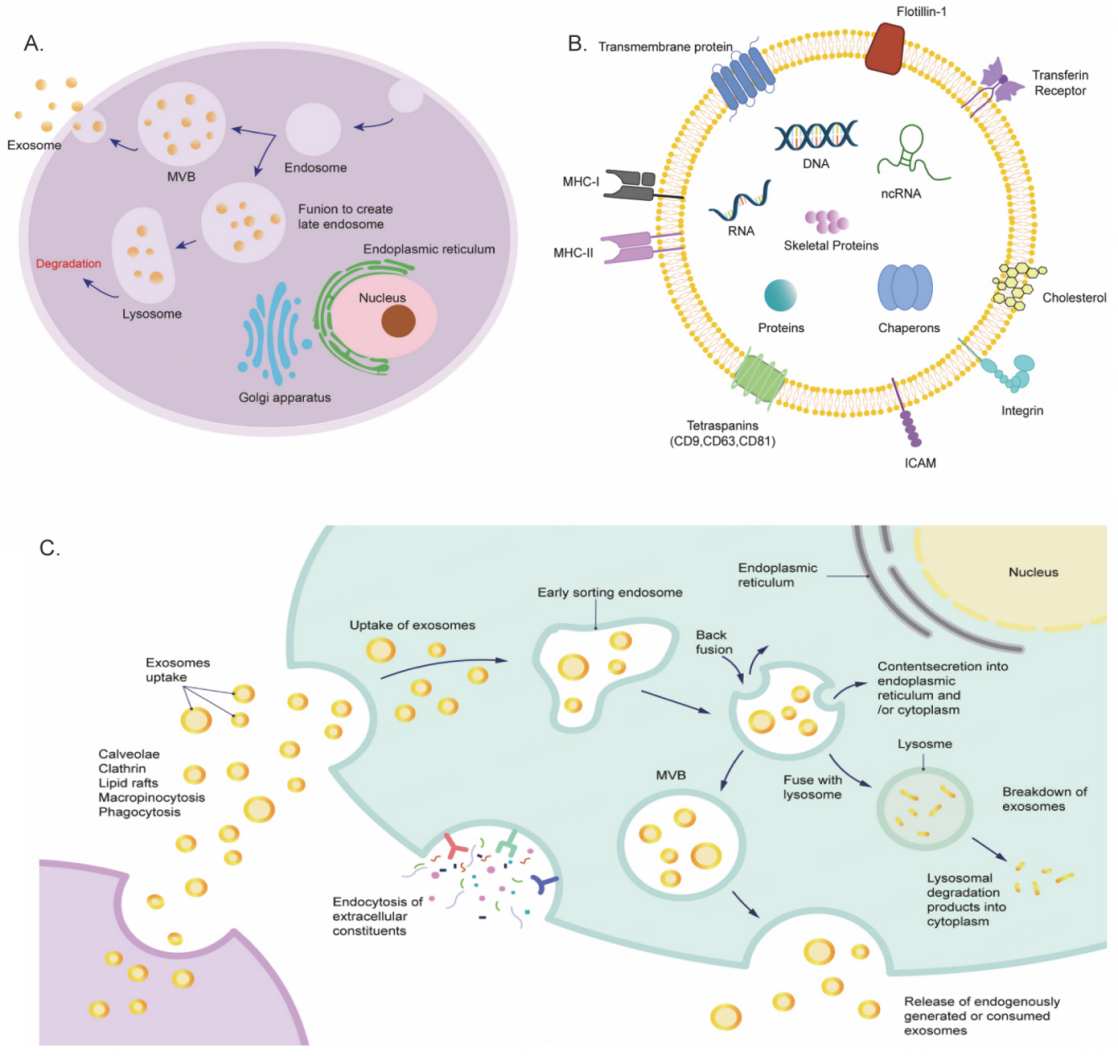
Biogenesis, structure, and action mode of the exosome. (A) Exosome formation is a function of endocytic membrane invagination and intraluminal vesicles (ILV) formation inside cells. Early maturation of endosomes leads to the formation of MVBs which are then delivered to lysosomes to be degraded or cross through microtubules to be combined with the plasma membrane and release exosomes into the extracellular space. (B) Exosomes contain various substances, such as mRNAs, miRNAs, proteins, enzymes, lipids, and carbohydrates. The exosome surface is decorated with various membrane proteins responsible for pathophysiological functions. (C) Exosomes facilitate communication between cells through three primary mechanisms. Signaling molecules act directly on the membrane's surface, regulate membrane fusion and its contents, and control the release of bioactive substances.

Extracellular vesicles (EVs) are membrane-bound vesicles that are released by cells into the extracellular space^[19]^. These vesicles are formed through the endocytic pathway, where the cell membrane invaginates to form a vesicle that fuses with an early endosome. Over time, specific proteins bind to the membrane, causing further inward bending and the formation of smaller vesicles within the endosome, known as multivesicular bodies (MVBs). These MVBs then move along protein tracks towards the cell surface. Some MVBs are degraded in lysosomes, while others fuse with the cell membrane, releasing the vesicles contained within the MVBs^[20]^ [Figure 1A]. EVs can be classified based on their size, according to the “MISEV2018 guideline”. Exosomes (EXOs) are typically 30-120 nm in diameter, microvesicles (MVs) or ectosomes or microparticles range from 0.1-1.0 μm, and apoptotic bodies (ABs) are larger, ranging from 0.8-5.0 μm^[21,22]^. Among these, exosomes are of particular interest as they carry a diverse range of proteins, lipids, DNA, RNA, and other important molecules for transporting substances and transferring information between cells^[23]^ [Figure 1B]. Exosomes can enter recipient cells through endocytosis or by binding to specific receptors on the cell membrane, releasing their contents into the recipient cell and participating in various biological processes^[24]^ [Figure 1C].

The origin and composition of exosomes determine their specific functions. For instance, exosomes derived from reticulocytes, which are immature red blood cells, are enriched with transferrin receptors (TfR or CD71) due to the selective removal of proteins during their maturation into erythrocytes^[25-27]^. Mesenchymal stem cells (MSCs), which can be derived from various tissues or cells such as the umbilical cord, placenta, adipose tissue, body fluids, and induced pluripotent stem cells (iPSCs), also exhibit different functions and properties depending on their source. For example, exosomes derived from human adipose tissue MSCs (ASCs) have shown higher renal protease activity, suggesting their therapeutic potential for Alzheimer’s disease. Exosomes derived from human iPSC MSCs (iMSCs) have been found to alleviate symptoms of osteoarthritis (OA). Additionally, exosomes derived from human bone marrow MSCs (BM-MSCs) have been shown to reduce cell proliferation and induce apoptosis, while ASC-derived exosomes increase proliferation but do not induce apoptosis in U87MG glioblastoma cells. Exosomes derived from tumor cells play a role in tumor growth, metastasis, and immunomodulation. For example, tumor-derived exosomes have been found to promote bone metastasis in non-small cell lung cancer and influence macrophage polarization^[28]^.

## EXOSOME-BASED DRUG DELIVERY SYSTEM

### Limitations of current approaches

Targeted drug delivery is a major focus in drug development, and the design and development of drug carriers play a crucial role in this process. It has been demonstrated that selecting an appropriate drug carrier based on the characteristics of the drug can improve its physical and chemical properties and enhance its availability. Currently, viral and non-viral vectors are the main drug carriers used in clinical and medical trials^[29]^. However, both types of vectors have their limitations. Viral vectors, such as adenovirus, herpes simplex virus, and adeno-associated virus, have low specificity, limited safety profiles, inadequate targeting capabilities, and potential carcinogenicity, which hinder their clinical application^[30]^. On the other hand, non-viral vectors, such as nanoparticle vectors, are limited by the toxicity of the raw materials used, the availability of suitable materials as vectors, and the low efficiency of drug encapsulation^[31]^. In comparison to other extracellular vesicles, exosomes have the advantage of being smaller in size, which allows them to penetrate tissue barriers more effectively, including the blood-brain barrier and blood-thymus barrier. The blood-brain barrier, for example, allows particles below 200 nm in size to pass through freely, without considering factors such as shape, composition, or stiffness^[22]^. However, this smaller size of exosomes may present challenges in terms of limited drug loading capacity.

### Advantages of exosome-based drug delivery system

Exosomes, with a diameter of less than 200 nm, have the ability to evade rapid clearance by mononuclear phagocytes and can penetrate various biological barriers with enhanced osmotic retention effects^[12,13,32]^. As drug carriers, exosomes offer significant advantages: Good biocompatibility and non-immunogenicity; therefore, exosomes have a reduced likelihood of *in vivo* immune rejection, improving drug utilization^[10]^. High stability in blood: Exosomes possess a deformable cytoskeleton and a gel-like cytoplasm-derived core, allowing them to resist external crushing and damage, thereby maintaining their structural integrity. This protective feature ensures the safe transport of encapsulated drugs to specific tissues or organs^[11]^. Interaction with receptor cells: Exosomes can interact with target cells by binding to adhesion molecules and carrier ligands on the cell membranes, such as tetraspanin, integrin, CD11b, and CD18 receptors^[33]^. This interaction facilitates the transport of cargo to the desired cells. Abundance and cargo diversity: Exosomes are naturally rich in mRNA, miRNA, non-coding RNA, lipids, proteins, and metabolites^[34]^. This abundance makes exosomes potential carriers for drug transport. For example, cardiomyocyte-derived exosomes were found to contain mRNA and DNA that promote metabolism-related activities^[35]^. Exogenous RNA can be loaded onto exosomes through mixed incubation and electroporation. Encapsulated plasma membrane with a hydrophilic core^[36]^: The structure of exosomes allows them to act as carriers. The surface molecules of exosomes can self-modify and adapt to confer cellular and tissue specificity. Hydrophobic therapeutic agents can be loaded directly onto the exosome membrane, while hydrophilic therapeutic agents can be loaded into the hydrophilic core of the exosome. Additionally, exosomes can be easily labeled and tracked *in vivo* using techniques such as transmission electron microscopy and differential scanning calorimetry, facilitating the observation of drug delivery^[36-38]^.

## EXOSOME-BASED DRUG DELIVERY METHODS

### Traditional exosome-based drug delivery method

Exosomes, with their natural phospholipid bilayer membrane structure, provide a means to incorporate lipophilic drugs, which can be released slowly from the membrane, resulting in sustained drug release. The method of drug carriage by exosomes depends on the hydrophilic or hydrophobic nature of the drug. There are two main methods of exosome carriage: First, active transport: This method involves techniques such as ultrasound, extrusion, and electroporation to actively load the drug into the exosomes. These techniques disrupt the exosome membrane, allowing the drug to be incorporated into the exosome’s lipid bilayer. Second, passive carriage: In this method, the drug is mixed directly with the exosomes or co-cultured with exosome donor cells. The drug is then naturally taken up by the exosomes during their biogenesis process^[39]^. This passive method does not require any external manipulation of the exosomes [Table 1].

**Table 1:** The exosome-based drug-carrying method

	**Method**	**Mechanism**	**Merits**	**Demerits**
Active	Sonication	Additional mechanical shear forces weaken the integrity of the outer body membrane with ultrasound^[44]^	High loading efficiency	Disruption of exosome integrity;Causes exocrine remodeling/deformation
	Surfactant treatment (e.g., Saponin)	Co-cultured with saponin solutions and then purified by dialysis membranes and chromatographic columns	Directly Incorporation of proteins; High loading efficiency	Hemolytic activity
	Extrusion	Mixing of target drugs with crushed exosome membranes using an extruder with nanoporous membranes^[45]^	High loading efficiency; uniform particle size	Possible changes of the exosome membrane and the zeta potential of the original exosomes
	Freeze-thaw cycles	Repeatedly frozen at -80°C or lower (liquid nitrogen), then thawed at room temperature^[46]^	High loading efficiency; Simple	Aggregation of exosomes
	Electroporation	Short high voltage electrical pulses form micropores in the exosome membrane	Loading with large molecules and smaller hydrophilic molecules	the risks of RNA and exosomes aggregation
	Chemical transfection	Gene edition^[47]^	Stability; high loading efficiency for peptides, proteins, and nucleic acids	Time and financial consumption; hard to quantitation; poor controllability
Unactive	Direct ncubation of drugs with exosomes	Different drug concentration gradients	Simple	Low loading efficiency
	Mixing with exosome donor cells after co-incubation	Cells cytosolicize the drug and then secrete it exosomes loaded with drugs	Simple	Low oading efficiency and low output drug concentration
Targeted delivery methods	Engineered exosomes	Genetic engineering modification of extracellular surface proteins^[43]^	Convenient, simple, and targeted	Possible changes to the protein alteration

### Targeted drug delivery method: engineered exosomes

The potential of exosomes as drug carriers is greatly enhanced by their ability to load drugs and achieve targeted transport and tracking through surface modifications. When exosomes enter the body, they are rapidly cleared by immune cells or accumulate in excretory organs such as the liver, spleen, and lungs^[40]^. The efficacy of a drug is closely related to its retention at the site of disease. Therefore, research on how to ensure that drug-loaded exosomes reach their intended target areas has become a hot topic. To achieve targeted delivery, researchers have developed engineered exosomes as cell-specific delivery vehicles^[41]^. There are two main approaches to modify the surface of exosomes^[42,43]^ [Table 1^[43-47]^]: Genetic engineering: Exosome donor cells are genetically modified to express specific peptides and proteins on the surface of exosomes. These modifications allow for cell-specific targeting and enhance the delivery of exosomes to desired cells. Chemical modification: Targeting ligands are anchored to the exosome membrane using splicing reactions or lipid assembly. This method involves attaching specific molecules to the exosome surface, enabling targeted delivery to specific cells or tissues. Both genetic engineering and chemical modification methods modify the surface molecules of exosomes to achieve cellular and targeting modifications, enhancing their ability to deliver drugs to specific sites.

Engineering modification of exosomes is a convenient method to introduce new properties to exosomes^[48]^. Once engineered exosomal exoproducts are obtained, the targeting efficiency of the modified exosomes can be evaluated. This can be done by labeling the engineered exosomes or their surface proteins with fluorescent dyes. The labeled exosomes can then be injected into mice via the tail vein, and their biodistribution in different tissues can be visualized using fluorescence microscopy^[48,49]^. It is important to note that the quality of exosomes obtained through different loading methods, as well as the drug loading rate and stability, can vary. Different loading methods may result in differences in the characteristics and performance of the loaded exosomes. Therefore, it is crucial to carefully evaluate and optimize the loading methods to ensure the desired drug loading efficiency and stability of the exosomes.

After loading of drugs onto exosomes, transmission electron microscopy (TEM) was used to observe the morphology of exosomes, dynamic light scattering (DLS) was applied to measure the size distribution of exosomes, exosome signature markers (CD9, Alix, Tsg101, and Calnexin) were detected by immunoblotting^[21,50]^, and qRT-PCR was performed in order to assess the amount of drugs in exosomes^[51]^. By employing these techniques, researchers can gain insights into the morphology, size distribution, protein composition, and drug loading efficiency of the loaded exosomes. These characterizations are crucial for evaluating the quality and performance of drug-loaded exosomes.

## APPLICATION OF EXOSOME-LOADED DRUG DELIVERY SYSTEM IN DIABETES AND ITS COMPLICATIONS

The treatment for DM involves the administration of oral hypoglycemic drugs and insulin injections. Patients diagnosed with type 1 diabetes mellitus (T1DM) require multiple daily insulin injections, which can potentially lead to severe hypoglycemia and give rise to symptoms such as anxiety, fatigue, and loss of consciousness^[52]^. On the other hand, patients with type 2 diabetes mellitus (T2DM) may experience side effects from traditional medications. For instance, metformin can cause indigestion, diarrhea, and heartburn^[53]^, while Dipeptidyl peptidase-4 (DPP-4) inhibitors can increase the risk of heart failure^[54]^. Furthermore, complications associated with diabetes, such as slow wound healing, pose significant challenges in terms of treatment. Despite recent advancements in the development of new drugs, the effectiveness of targeting specific growth factors and cytokines remains unsatisfactory due to issues such as chemical instability and susceptibility to degradation^[55]^. Consequently, there is a growing focus on the development of novel drugs and therapeutic approaches in diabetes research.

**Figure 2:**
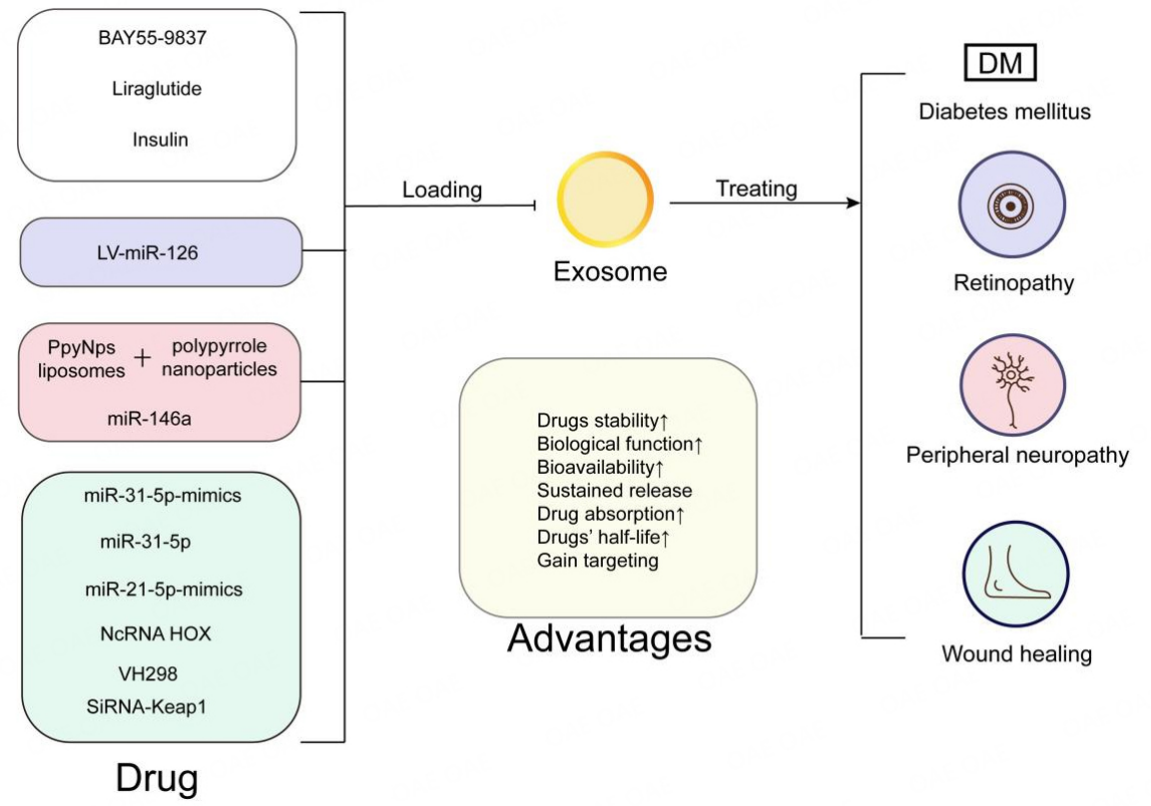
The advantages of exosome-based drug delivery therapy in diabetes mellitus and its complications. Exosome-based drug delivery systems can improve diabetic complications, including retinopathy, peripheral neuropathy, and delayed wound healing, by transporting drugs.

Numerous experiments have provided evidence that the selection of appropriate drug carriers, based on the properties of the drugs, can improve their physicochemical characteristics and enhance drug utilization^[56]^. Considering the advantages of exosome-based drug delivery systems mentioned earlier, we believe that they hold significant potential for the treatment of diabetes and its complications. However, there are only a limited number of reports on the application of exosome-based drug delivery in the treatment of diabetes. The potential application of exosome-based drug delivery systems in the treatment of diabetes and its associated complications has been shown in Figure 2.

### Diabetes

#### Exosomes protect insulin from proteolytic degradation, preserve its natural structure and biological function and improve its bioavailability

Diabetes is characterized by high blood sugar levels resulting from issues with insulin secretion or function. Insulin, a hormone that helps regulate blood sugar levels, can be degraded by enzymes in the digestive tract. However, exosomes, small lipid-based structures, have the ability to protect insulin from degradation and maintain its function^[57]^. In experiments conducted by Rodríguez-Morales B and colleagues, insulin was labeled with an isothiocyanate fluorophore. This labeled insulin was then loaded into exosomes derived from various cell types, including hepatocellular carcinoma cells, primary skin fibroblasts, and brown mouse insulinoma epithelial cells, using electroporation. These insulin-loaded exosomes were taken up by their respective parent cells and were found to enhance glucose transport and metabolism in a hyperglycemic environment *in vitro*^[58]^. These findings suggest that exosome-based delivery of insulin has the potential to improve glucose regulation in diabetes.

In addition to their ability to protect insulin from degradation, exosomes have several advantages over synthetic nanocarriers as drug transporters. Exosomes are natural products that reduce the risk of toxicity in recipient cells and clearance by the immune system. Moreover, drug-loaded exosomes have been shown to be stable and capable of preserving the original biological properties of the drug for extended periods, such as up to 6 months at -80 °C^[59]^. These characteristics make exosomes stable, biocompatible, and non-cytotoxic vehicles for insulin delivery. These advantages further support the potential of exosome-based drug delivery systems for the treatment of diabetes.

#### Engineered exosomes extend the half-life of the drug, improve its glucose (GLC) responsiveness in the blood and allow it to gain targeting

Research has demonstrated that vasoactive intestinal peptide receptor type 2 (VPAC2) agonists can enhance glucose-dependent insulin secretion. However, the VPAC2 agonist BAY55-9837 has limitations such as poor stability in aqueous buffers and a short duration of action *in vivo*, making it unsuitable for long-term diabetes treatment. To overcome these challenges, Zhuang *et al*. developed a carrier system by loading BAY55-9837 into reticulocyte exosomes and modifying superparamagnetic iron oxide nanoparticles (SPIONs) with transferrin^[60]^. The SPIONs were then bound to transferrin receptors on the surface of the reticulocyte exosomes, resulting in the self-assembled BAY-exosome-SPION carrier system. This system extended the plasma half-life of BAY55-9837 and facilitated rapid drug accumulation in pancreatic β-cells under external magnetic guidance. As a result, the glycemic response to BAY55-9837 was enhanced, leading to increased insulin secretion and improved control of hyperglycemia *in vivo*. This innovative approach holds promise for the development of more effective and stable therapies for diabetes.

#### Exosomes enhance the bioavailability of therapeutic peptides

In a study conducted by Xu *et al.*, the anti-diabetic drug liraglutide was loaded into lactogenic exosomes called mEXOs (LRT-EV). The researchers found that the sublingual route of administration effectively reduced blood glucose levels, and they compared various dosing regimens. Furthermore, they observed that liraglutide loaded into mEXOs had up to 10% higher bioavailability compared to the drug alone (*in vivo*)^[61]^. Additionally, other miRNAs, such as miR-375-3p and miR223, have been implicated in the regulation of β-cell function^[62]^. Although their use as drugs loaded into exosomes has not been reported yet, this discovery could potentially lead to breakthroughs in therapeutic approaches by incorporating them into exosomes. This suggests that exosome-based delivery systems have the potential to improve the efficacy and bioavailability of anti-diabetic drugs.

### Diabetic wound healing

Diabetes-related amputations often occur due to impaired wound healing. The process of wound healing involves a complex interplay of various cells, cytokines, and extracellular matrix components, and it typically progresses through three phases: inflammation, proliferation, and remodeling^[63]^. Studies have shown that dysregulation of specific molecules, such as miRNAs, lncRNAs, and siRNAs, can contribute to delayed wound healing in diabetic patients^[64-68]^. These dysregulated molecules can be present in elevated or decreased levels in the patient’s body, leading to impaired wound repair. Furthermore, diabetic wounds are often slow to heal and have a high rate of recurrence, making treatment with growth factors and cytokines alone insufficient. Therefore, there is a need for innovative approaches, such as exosome-based therapies, to enhance the wound healing process and improve outcomes for diabetic patients with impaired wound healing.

#### Exosomes enhance miRNA stability and cellular uptake

Studies have demonstrated that miR-31-5p is downregulated in diabetic wounds and plays a crucial role in angiogenesis^[68]^. MicroRNAs (miRNAs) are non-coding RNAs that regulate gene expression. The interaction between miRNAs and their target genes is complex and influenced by various factors, including subcellular localization, abundance, and affinity of the miRNA-mRNA interaction^[69]^. Research has shown that physical exercise and specific dietary components can modulate miRNA expression in *in vitro* and *in vivo* models^[70]^. However, free miRNAs are susceptible to degradation in the external environment, and their uptake by cells is hindered by their high molecular weight and negative charge, which leads to repulsion from the cell membrane. In contrast, exosomes, with their protective membrane structure, serve as ideal carriers for miRNA delivery^[71]^. Yan C’s team loaded a miR-31-5p mimic (mECO-31) into mEXOs using electroporation. The mECO-31-mEXOs significantly reduced the expression of hypoxia-inducible factor 1α subunit inhibitory factor (HIF1AN), improved endothelial cell function, and accelerated the healing process of diabetic wounds. The mEXOs enhanced miRNA retention and permeability in the wound environment (*in vivo*)^[72]^. Additionally, Huang *et al*. engineered exosomes derived from human embryonic kidney cells based on miR-31-5p. These exosomes promoted rapid closure of diabetic wounds through pro-angiogenesis, pro-fibrogenesis, and re-epithelialization in both *in vitro* and *in vivo* models^[73]^. These findings highlight the potential of exosome-based therapies for enhancing wound healing in diabetic patients.

Indeed, engineered exosomes have emerged as promising carriers for miRNAs, addressing challenges such as immune response, unstable extracellular environment, low bioavailability, and barriers to cellular uptake^[73]^. Lv *et al*. successfully loaded miR-21-5p mimics into exosomes derived from human adipose stem cells (hASC-exos). The miR-21-5p-hASC-exos promoted diabetic wound healing by enhancing keratinocyte proliferation and migration through mechanisms such as re-epithelialization, collagen remodeling, angiogenesis, and vascular maturation (*in vivo*)^[74]^. These findings highlight the potential of exosomes to protect miRNAs from degradation in the wound microenvironment and facilitate their therapeutic effects in promoting wound healing in diabetic patients.

#### Exosomes enhance the expression of long non-coding (lncRNAs) in cells

The upregulation of the long non-coding RNA (lncRNA) HOX transcriptional antisense RNA (HOTAIR) has been found to positively correlate with angiogenesis induced by endothelial cell-derived extracellular vesicles (EVs). Conversely, reduced expression of HOTAIR has been shown to impact diabetic wound healing outcomes. In a study, researchers loaded HOTAIR into extracellular vesicles derived from bone marrow mesenchymal stem cells (MSCs) and found that these HOTAIR-MSC EVs significantly increased HOTAIR levels, leading to improved wound healing and vessel growth in diabetic mice (*in vivo*)^[75]^. These findings suggest that HOTAIR-MSC EVs could serve as a therapeutic tool to enhance wound repair and promote angiogenesis in diabetic patients.

#### Exosome and gelatin methacryloyl (GelMA) combination enables sustained release, high bioavailability, and targeted delivery

Researchers have made an exciting discovery regarding the use of extracellular vesicles (EVs) loaded with a small-molecule compound called VH298 for healing diabetic wounds. Wang *et al*. developed a wound dressing called Gel-VH-EV, which combines a functionalized cellular exosome loaded with VH298 and GelMA hydrogel^[76]^. This dressing was tested *in vivo* and showed promising results in improving wound recanalization and enhancing local blood supply in mice with bronchial disease. The Gel-VH-EV dressing exhibited favorable mechanical properties and sustained-release capabilities, making it a practical option for promoting optimal wound healing. This research highlights the potential of utilizing EVs and small-molecule compounds for advanced wound healing therapies.

#### Exosomes protect siRNA from degradation and enhance drug absorption

Excessive and uncontrolled oxidative stress plays a critical role in the development of diabetic wounds. Previous studies have demonstrated that reducing the expression of Kelch-like ECH-associated protein 1 (Keap1) through siRNA therapy can alleviate oxidative stress damage in diabetic wounds. However, siRNA faces limitations such as its negative charge, instability in circulation, immunogenicity, and the toxicity of available siRNA vectors. To overcome these challenges, Xiang *et al.* loaded and delivered siRNA-Keap1 (siKeap1) to modified exosomes (mEXOs-siKeap1) using sonication^[77]^. This approach protected the siRNA from degradation in the gastrointestinal tract, enhanced its efficacy, and accelerated the healing of diabetic wounds. Furthermore, this treatment resulted in increased collagen formation and neovascularization in both *in vitro* and *in vivo* mouse models of diabetes. These findings suggest that mEXOs-siKeap1 could be a promising therapeutic strategy for promoting diabetic wound healing by targeting oxidative stress.

### Diabetic peripheral neuropathy

Diabetic peripheral neuropathy (DPN) is a condition that affects a significant proportion of adult diabetic patients, with a prevalence rate ranging from 10% to 26%^[78]^. Researchers have investigated various signaling pathways associated with DPN, including the late glycosylation end products, protein kinase C pathway, oxidative stress pathway, and autophagy. However, clinical outcomes in the treatment of DPN have been largely disappointing^[79,80]^. DPN is characterized by neuronal cell damage induced by persistent hyperglycemia, reduced neurovascular flow and neuronal ischemia resulting from chronic hyperglycemia, and the production of adhesion molecules by immune cells and adipocytes, as well as inflammatory cytokines triggered by hyperglycemia and dyslipidemia. Chronic inflammation associated with diabetes can also contribute to neurovascular damage, including axonal degeneration, endothelial dysfunction, and ongoing neuronal injury^[81]^. These factors collectively contribute to the development and progression of DPN. Further research is needed to better understand the underlying mechanisms and develop effective therapeutic strategies for managing this debilitating condition.

#### Engineered exosomes efficiently transfer current at the sciatic nerve

Electrical stimulation has been shown to be beneficial for nerve repair and regeneration in DPN^[82]^. It can increase the expression of growth factors such as vascular endothelial growth factor (VEGF) and nerve growth factor (NGF), promoting angiogenesis, reducing inflammation, relieving neuropathic pain, and improving nerve regeneration. However, there is a need for more selective and effective electrical stimulation therapies. To address this, Singh A and colleagues developed a conductive exosome system (DCES). This system combines exosomes derived from bone marrow mesenchymal stem cells (BMSCs) with polypyrrole nanoparticles encapsulating PpyNps liposomes through a freeze-thaw process. The DCES can efficiently deliver electrical currents to the sciatic nerve and transport regenerative molecules to specific injury sites by expressing tissue-specific targeting components on exosomes, as demonstrated in *in vivo* experiments^[83]^. This innovative approach has shown promise in reversing nerve tissue damage associated with DPN. The use of the DCES system provides a potential strategy for targeted and effective electrical stimulation therapy in DPN, offering new possibilities for nerve repair and regeneration in diabetic patients. Further research and clinical studies are needed to validate its efficacy and safety in human subjects.

#### Engineered exosomes enable the enhanced effect of miR-146 treatment

Recent research has shown that miR-146a has the potential to reduce inflammation and alleviate DPN. However, there are challenges that need to be addressed before miR-146a can be used clinically. These challenges include its vulnerability to degradation, low stability in serum, and the potential for off-target effects and immune responses. To overcome these challenges, Fan B and colleagues developed an engineered MSC exosome (Exo-146a) that carries miR-146a using chemical transfection technology. Compared to native MSC-exosomes, Exo-146a demonstrated more potent anti-inflammatory and protective effects on neurological function and promoted neurological recovery in DPN mice *in vivo*. Additionally, Exo-146a shortened the duration of treatment and achieved better recovery outcomes^[84]^. The development of Exo-146a as a delivery system for miR-146a provides a promising approach for addressing the limitations of miRNA-based therapies in DPN. Further research and clinical studies are needed to validate the efficacy, safety, and long-term effects of Exo-146a in human subjects.

### Exosomes can enhance the therapeutic effect of miR-126 in diabetic retinopathy

Diabetic retinopathy (DR) is a common complication of diabetes mellitus (DM) characterized by damage to the retinal microcirculation due to chronic hyperglycemia^[85]^. DR can lead to severe consequences, including proliferative neovascularization, which often results in proliferative DR and irreversible vision loss^[86]^. Current treatment options for DR include laser therapy, vitrectomy, and anti-vascular endothelial growth factor drugs. However, these treatments carry the risk of nerve damage and retinal atrophy. Therefore, there is a need to explore alternative therapeutic approaches for DR. In a study by Pan Q and colleagues, miR-126 was transfected with a miR-126 mimic or miR-126 short hairpin RNA to obtain miR-126-overexpressing MSC-derived exosomes (MSC-EXsmiR-126) and miR-126 knockdown MSC-derived exosomes (MSC-EXsSimiR-126). These exosomes were then intravitreally injected with MSC-derived exosomes. The study found that MSC-derived exosomes enhanced the survival and angiogenic function of endothelial cells (ECs) injured by hypoxia/reoxygenation (H/R) by delivering miR-126 to the ECs. Furthermore, MSC-derived exosomes reduced hyperglycemia-induced endothelial dysfunction and inflammation in the retina by regulating the expression of high-mobility group box 1 and inhibiting nuclear factor-κB/p65 activation, as demonstrated *in vitro*^[87]^. These findings suggest that MSC-derived exosomes carrying miR-126 have the potential to improve endothelial function and reduce inflammation in the retina, offering a potential therapeutic approach for DR. Further research is needed to validate these findings in animal models and clinical trials.

### Exosomes containing miRNAs improve insulin sensitivity and reduce the inflammatory response

Recent studies have confirmed the relationship between exosomes, obesity, and insulin resistance. In obese mice, adipose tissue macrophages secrete exosomes containing miRNAs that can induce glucose intolerance and insulin resistance in lean mice^[88]^. Conversely, exosomes derived from Adipose-Derived Stem Cells (ADSCs) promote insulin sensitivity and reduce inflammation by inducing an anti-inflammatory M2 phenotype. This suggests that exosomes play a role in immune and metabolic homeostasis. The miRNAs carried by these exosomes mainly regulate TGF-β and Wnt/β-catenin signaling, which are crucial in the development and progression of chronic inflammation^[89]^. Exosomes released by adipose tissue in obese mice are taken up by mononuclear cells, leading to their differentiation into active macrophages with increased release of pro-inflammatory cytokines such as TNF-α and IL-6. This exosome-mediated activation involves the TLR4/TRIF pathway and may contribute to obesity-associated insulin resistance^[90]^. These findings highlight the role of exosomes in mediating the crosstalk between adipose tissue, inflammation, and insulin resistance. Further research is needed to fully understand the mechanisms underlying these processes and to explore the potential therapeutic applications of exosomes in obesity and insulin resistance.

## CHALLENGES OF EXOSOME-BASED DRUG DELIVERY SYSTEM

There are still numerous issues with exosome-based drug delivery systems. The research is focused on acquiring high-quality and active exosomes. A summary of the current exosome extraction methods has been compiled in Table 2^[91-94]^ for comparison. One of the major challenges associated with exosomes is the requirement for a scalable production method that can generate them in large quantities with specific desired effects^[95,96]^. Regrettably, none of the existing exosome extraction techniques fully meet these ideal criteria. Additionally, there is currently no purification technique that combines high purity and scalability, highlighting the need for the development of an effective method for exosome purification^[97]^. The detection and identification of exosomes also pose significant concerns^[96]^. It is crucial to accurately assess whether the isolated particles are indeed exosomes and to determine their purity levels^[98,99]^. Studies have demonstrated the promising potential of exosome-based drug-loading systems for clinical applications^[100]^. However, most drugs are still in the early stages of *in vivo* and *in vitro* experimentation, and their translation to the clinical stage remains a significant hurdle. Several obstacles, including large-scale production, stable preparation, storage solutions, and quality control, need to be addressed before clinical implementation can be achieved. Advancements in the development of cell-derived engineered exosomes, as well as improvements in isolation, purification, and drug-loading technologies, will play a crucial role in overcoming these challenges^[101]^.

**Table 2:** Comparison of current exosome extraction techniques

**Methodology**	**Mechanisms**	**Advantages**	**Disadvantages**
Ultracentrifugation	Sequential separation based on density and particle size^[82]^	Simple handling and a high number of vesicles obtained	a time-consuming process; low purity; high equipment cost; Disruption of exosome integrity
Density gradient centrifugation	Ultracentrifugation combined with sucrose density gradients^[82]^	High purity of the obtained exosomes	Cumbersome and time-consuming steps
Ultrafiltration	Separation and extraction of different particle size^[83]^	Simple operation, timesaving, cost-efficient operation, high output, and efficiency	the pressure and shear forces during filtration deform and damage the exosomes
Magnetic-activated cell sorting(MACS)	Separation of labelled cells bound to magnetic beads using a magnetic field^[84]^	High specificity and ease of handling	Low efficiency and susceptibility of exosome bioactivity to pH and salt concentration
Separation of exosomes by sieving	Exosomes are sieved out of the sample through the membrane using pressure or electric fields	Short separation times and high purity	Low recovery rate
Size exclusion chromatography	Separation of molecules of different sizes and numbers using molecular sieves^[83]^	Precise separation of small and large molecules	Time-consuming, not suitable for large sample sizes
Polymer-based precipitation technology	Polyethylene glycol (PEG) binds to hydrophobic proteins and lipid molecules for co-precipitation^[85]^.	Low impact on isolated exosomes	Low purity and recovery, high levels of heterogeneous proteins, uneven particle size, destruction of exosomes
Reagent Kits	Multiple	No special equipment is required, simple operation, high purity, and high recovery of exosomes	Just starting to develop, less variety and high cost

## CONCLUSION

In this review, we provide an overview of exosome-based drug delivery methods, their potential applications in treating diabetes and its complications, and the challenges associated with exosomal drug delivery systems. Preliminary studies have demonstrated the strong therapeutic potential of exosomal drug delivery systems. However, it is important to acknowledge that this field of research is still relatively young and rapidly evolving, with numerous ongoing studies. Accumulation of exosomes at the site of injury has been observed in many studies^[102]^. In recent years, significant attention has been focused on the development of “engineered exosomes”^[43]^, where scientific interventions are employed to modify the properties of exosomes to achieve specific therapeutic objectives. These modifications may involve genetic alterations of the exosome’s parent cell or chemical modifications of the exosome’s surface molecules. These strategies can enhance the targeting ability of exosomes, their capacity to carry and release drugs, or their interaction with receptor cells^[102]^. However, after the modification of exosomes, it is essential to conduct thorough testing to evaluate potential issues such as loss or alteration of the original exosome contents and the introduction of unwanted substances.

Engineered exosomes have demonstrated promising clinical potential in various fields, including tumor, cardiovascular diseases, and neurological diseases, and have shown improved therapeutic effects and targeting compared to natural exosomes^[48,103]^. However, the clinical translation of engineered exosomes faces several challenges. Variations in the number of cell generations and culture sera used to derive exosomes can lead to differences in exosome characteristics. Different extraction methods can also impact the yield and homogeneity of exosome subpopulations. Standardization of cell culture scale-up, isolation, and extraction processes is necessary. Additionally, there is a need to establish a gold standard for quantification and molecular and physical characterization of exosomes. Quality control procedures are required to ensure that exosome products meet key quality attributes. Currently, there is no consensus on how to test the safety and efficacy of engineered exosomes^[104]^. While no engineered exosomes have reached clinical translation yet, their therapeutic potential is evident. Despite challenges such as technical difficulties in large-scale preparation and purification, the need for validation in clinical applications, and cost and acceptability considerations, with further research and innovation^[105]^, exosome-based drug delivery systems hold promise as effective approaches to improve diabetes and its complications.

## DECLARATIONS

### Copyright

© The Author(s) 2023.
